# Shining a light on hematopoietic stem cells

**DOI:** 10.7554/eLife.81963

**Published:** 2022-08-19

**Authors:** Anne Schmidt

**Affiliations:** 1 Institut Pasteur, Department of Developmental and Stem Cell Biology, Université de Paris Cité, CNRS UMR3738, F-75015 Paris France

**Keywords:** hematopoietic stem cell, stem cell niche, microenvironment, correlative light, electron microscopy, dopamine beta-hydroxylase, serial section blockface scanning electron microscopy, Zebrafish

## Abstract

A combination of light and electron microscopy has revealed further details about the location and interactions of hematopoietic stem and progenitor cells.

**Related research article** Agarwala S, Kim KY, Phan S, Ju S, Kong YE, Castillon GA, Bushong EA, Ellisman MH, Tamplin OJ. 2022. Defining the ultrastructure of the hematopoietic stem cell niche by correlative light and electron microscopy. *eLife* **11**:e64835. doi: 10.7554/eLife.64835.

Blood cells perform a wide range of physiological functions in the body. Red blood cells transport oxygen from the lungs to various tissues around the body, while white blood cells protect the body against viruses, bacteria and other pathogens. However, all these different types of cells are produced by the same ‘mother cells’, the hematopoietic stem cells.

Remarkably, virtually all of the hematopoietic stem cells in adult vertebrate species arise during embryonic development (Dzierzak and Bigas, 2018). At first, these cells and their daughter cells – the hematopoietic stem and progenitor cells or HSPCs – all reside in specific developmental hematopoietic niches, before moving to different niches during adulthood.

In mammals, this niche is primarily localized in the fetal liver during development, before shifting to the bone marrow in adults. The niche in the bone marrow is sub-compartmentalized, with some hematopoietic cells residing near endothelial tissues, and others residing near bone tissues (Pinho and Frenette, 2019). However, less is known about the structure and organization of the niche in the fetal liver (Khan et al., 2016; Lewis et al., 2021). Now, in eLife, Owen Tamplin, Mark Ellisman and colleagues – including Sobhika Agarwala and Keun-Young Kim as joint first authors – report how they have combined different forms of microscopy to reveal new details of the niche during development (Agarwala et al., 2022). The experiments were performed on zebrafish, which is widely used as a model organism because it is transparent.

In the zebrafish embryo, hematopoietic stem cells migrate from the dorsal aorta to a region in the tail that is considered to be functionally equivalent to the fetal liver in mammals (Figure 1A; Murayama et al., 2006). From there, both hematopoietic stem cells and progenitor cells travel to the thymus and the kidney marrow (which is the equivalent to the bone marrow in mammals).

**Figure 1.**
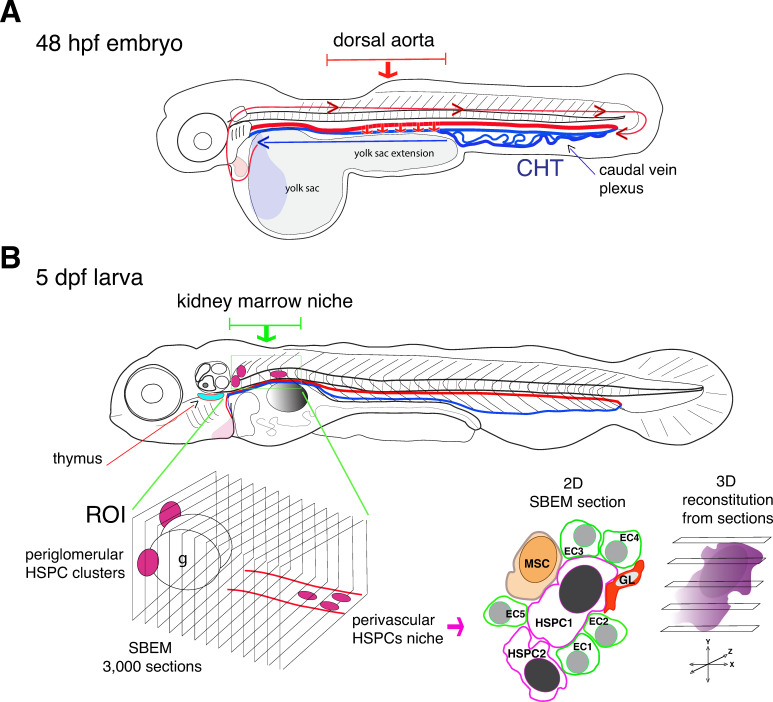
Visualizing hematopoietic stem cells in their developmental niches. (**A**) A zebrafish embryo 48 hours post fertilization (48 hpf) showing the dorsal aorta (thick red line) and vein (thick blue line) and the caudal hematopoietic tissue (CHT). Hematopoietic stem cells (HSCs) emerge from the dorsal aorta (downward red arrows) and then circulate via blood flow (blue and red lines with arrowheads), before settling in the CHT, where they start to multiply. (**B**) Subsequently, hematopoietic stem and progenitor cells (HSPCs) exit the CHT to settle into secondary niches, such as the thymus (turquois shape) and the kidney marrow (green rectangle; the grey round shape is the swim bladder). Agarwala et al. have used five-day-old larvae (5dpf) to visualize HSPCs in the diverse niches of the kidney marrow (ROI, region of interest; g, glomerulus), using live fluorescence microscopy. Automatic serial sectioning of the ROI generated about 3,000 sections, each of which was imaged at electron microscopic resolution (SBEM). Computer-assisted image treatment allowed to superposition fluorescence and electron micrographs images to identify HSPCs on 2D SBEM sections, to obtain high resolution 3D datasets (purple), and to visualize the entire HSPC contacting surfaces. This showed that in the perivascular region, HSPCs interact with contacting cells, such as ganglion-like cells (GL, red), a unique mesenchymal stromal cell (MSC, orange) and up to five endothelial cells (ECs).

Agarwala et al. developed workflows that first integrated fluorescent live imaging and light sheet microscopy to generate a three-dimensional visualization of the entire kidney region of zebrafish larvae. This enabled them to monitor HSPCs lodging deep in the kidney tissues. They then performed a series of sophisticated sectioning approaches on preserved specimens that led to 3D datasets of about 3,000 tissue sections, each coupled to high-resolution imaging at the sub-cellular scale.

The experiments revealed that the developing kidney encompasses various HSPC niches, each made of different combinations of cells (Figure 1B). In the anterior kidney region, HSPCs reside as clusters adjacent to the glomerulus, the filtering unit of the kidney; in the posterior vascular and perivascular region (the space surrounding the blood vessels), they are more dispersed. Moreover, HSPCs located exclusively in the posterior perivascular region are all in direct contact with a single stromal cell (a fibroblast-like cell that can differentiate into various cell types) and three quarter of them are in contact with ganglion-like cells that produce the neurotransmitter dopamine. When dopamine signaling was experimentally blocked, the number of HSPCs within the posterior region was reduced, confirming that the nervous system has a role in regulating HSPCs (Agarwala and Tamplin, 2018).

Otherwise, and irrespective of the localization, HSPCs are in contact with endothelial cells (between two to five cells per HSPC) and some 50–60% of them are in contact with a single stromal cell. Interestingly, this has also been observed in the tail region of the embryo (Tamplin et al., 2015). Some HSPCs are also in contact with red blood cells or other HSPCs.

These results suggest that a certain minimum of cellular components is needed to maintain the stemness potential of hematopoietic stem cells, at least between developmental niches. However, to validate this idea, one needs to also discriminate in situ between short-lived stem cells that are restricted to a developmental period, long-term stem cells that persist until the adult stage, and more differentiated progenitor cells (Dzierzak and Bigas, 2018). Moreover, the contacting cells, in particular endothelial and mesenchymal stromal cells, need to be characterized further (Zhang et al., 2021; Mabuchi et al., 2021). Achieving these aims will require spatially resolved genomics and proteomics, supported by new multiplexing technologies. Insights gained from these undertakings will help move the field of regenerative medicine towards the long-term goal of being able to reconstitute bona fide hematopoietic stem cells in the laboratory.
